# Broadband absorption and enhanced photothermal conversion property of octopod-like Ag@Ag_2_S core@shell structures with gradually varying shell thickness

**DOI:** 10.1038/s41598-017-18220-1

**Published:** 2017-12-19

**Authors:** Qian Jiang, Wenxia Zeng, Canying Zhang, Zhaoguo Meng, Jiawei Wu, Qunzhi Zhu, Daxiong Wu, Haitao Zhu

**Affiliations:** 1 College of Materials Science and Engineering, Qingdao University of Science and Technology, Qingdao, Shandong 266042 P. R. China; 2 College of Electromechanical Engineering, Qingdao University of Science and Technology, Qingdao, Shandong 266042 P. R. China; 3 College of Energy and Machenical Engineering, Shanghai University of Electric Power, Shanghai, 200090 P. R. China

## Abstract

Photothermal conversion materials have promising applications in many fields and therefore they have attracted tremendous attention. However, the multi-functionalization of a single nanostructure to meet the requirements of multiple photothermal applications is still a challenge. The difficulty is that most nanostructures have specific absoprtion band and are not flexible to different demands. In the current work, we reported the synthesis and multi-band photothermal conversion of Ag@Ag_2_S core@shell structures with gradually varying shell thickness. We synthesized the core@shell structures through the sulfidation of Ag nanocubes by taking the advantage of their spatially different reactivity. The resulting core@shell structures show an octopod-like mopgorlogy with a Ag_2_S bulge sitting at each corner of the Ag nanocubes. The thickness of the Ag_2_S shell gradually increases from the central surface towards the corners of the structure. The synthesized core@shell structures show a broad band absorption spectrum from 300 to 1100 nm. Enhanced photothermal conversion effect is observed under the illuminations of 635, 808, and 1064 nm lasers. The results indicate that the octopod-like Ag@Ag_2_S core@shell structures have characteristics of multi-band photothermal conversion. The current work might provide a guidance for the design and synthesis of multifunctional photothermal conversion materials.

## Introduction

Photothermal conversion (PTC) materials can absorb incident photons and generate heat. Hence they are applicable in many fields, such as photothermal therapy^1–5^, photothermal imaging^6,7^, solar energy harvesting^8,9^, sea water desalinization^10–13^, and so on. Different applications require specific optical absorption properties of PTC materials. For example, PTC materials applied in photothermal therapy are required to absorb near-infrared (NIR) rays which can penetrate biological tissues^14,15^. In contrast, PTC materials having strong absorption in the visible region are promising in solar energy harvesting and sea water desalinization for the reason that the highest irradiance of the solar spectrum is located at about 500 nm^16^. In recent years, many PTC materials have been designed and synthesized for specific applications. However, there is hardly any report on multi-functional PTC materials which can fulfil the requirements of different applications.

Broadband absorption is the first consideration in designing multi-functional PTC materials. Most PTC materials show strong absorption peaks at specific wavelengths. For example, the surface plasmon resonance (SPR) absorption peaks of gold and silver nanospheres are located at about 520 nm and 420 nm, respectively^17–19^. Sophisticated strategies have been developed to tune the absorption spectra of PTC materials. For example, Wang and his co-workers found that the longitudinal plasmon wavelengths of gold nanorods can be adjusted from 520 nm to 960 nm by changing the aspect ratios of the nanorods^20^. Then they demonstrated that broadband optical absorption can be achieved by mixing gold nanorods of different aspect ratios^21^. Tunable absorption spectra can also be achieved by adjusting the shell thickness of core@shell structures. For example, Oldenburg reported that the peak absorbance of SiO_2_@Au core@shell structures (120 nm cores) shifted from 550 nm to 800 nm when the shell thicknesses changed from 20 nm to 33 nm^22^. Li pointed out that the absorption peaks of SiO_2_@Au core@shell structures (50 nm cores) shifted from 730 nm to 1190 nm when the Au shell thickness varied from 12.5 nm to 2.5 nm^23^. Theoretically, broadband absorption can also be achieved by mixing core@shell structures with different shell thicknesses.

Although it is possible to tune the absorption spectra by synthesizing and mixing different nanostructures, it is still a big challenge to design and synthesize a specific nanostructure which exhibits broadband absorption properties. Herein, we propose a strategy to synthesize multi-functional PTC materials aiming at broadband absorption characteristics. Our target is a core@shell structure having gradually varying shell thickness. Given that a certain shell thickness leads to a corresponding absorption wavelength, a core@shell structure having gradually varying shell thickness may give broadband absorption spectrum. In the current work, Ag nanocubes with spatially different reactivity were applied as templates, which eventually resulted in gradually varying shell thickness in the synthesized Ag@Ag_2_S core@shell structures. Previously, Ag-Ag_2_S hybrids of different shapes including nanowires^24^, nanocubes^15,25^, triangular nanoplates^15^, and nanoprisms^26,27^ were synthesized via the sulfudition of the corresponding Ag nanocrystals. In those studies, the resulting hybrids possessed the same shapes as the Ag nanocrystals. In the current work, however, we demonstrate that Ag nanocubes can evolve into octopod-like Ag@Ag_2_S core@shell structures.

## Results

### Synthesis and characterization of the octopod-like Ag@Ag_2_S core@shell structures

**Figure 1:**
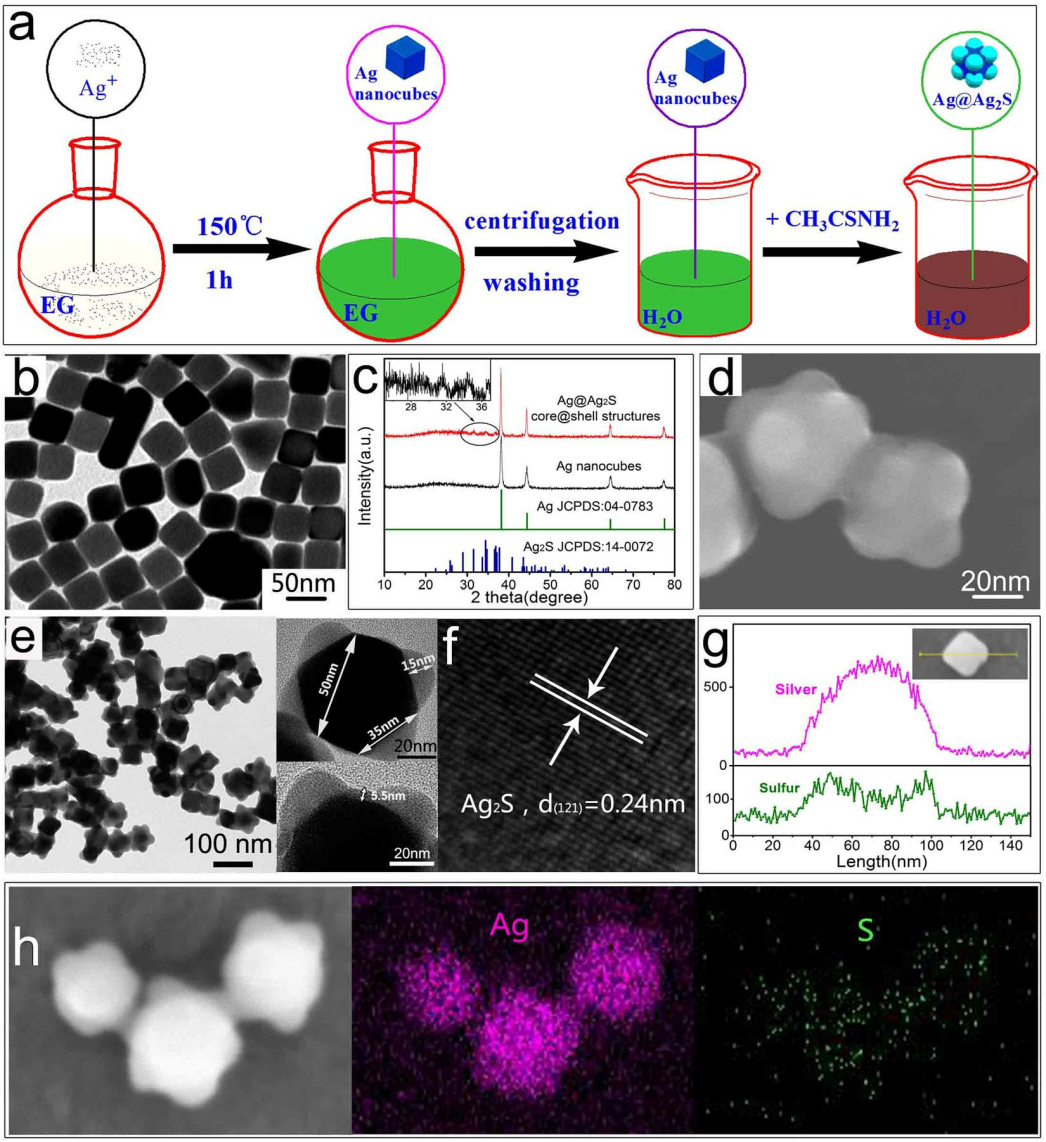
(**a**) Schematic illustration of the synthetic route for the Ag nanocubes and Ag@Ag_2_S core@shell structures, (**b**) TEM image of the Ag nanocubes, (**c**) XRD patterns of the products, (**d,e**) SEM and TEM images of the Ag@Ag_2_S core@shell structures, (**f**) HRTEM image of a bulge at the corner of the core@shell structure, (**g**) Elemental distribution of a core@shell structure, and (**h**) SEM image and the elemental mapping of the Ag@Ag_2_S core@shell structures.

As illustrated in Fig. 1a, CF_3_COOAg was reduced in ethylene glycol (EG) to prepare Ag nanocubes^28^. The resulting Ag nanocubes were then washed and dispersed in water to synthesize Ag@Ag_2_S core@shell structures through *in-situ* sulfidation with thioacetamide (TAA). The Ag nanocubes have well defined cubic shape with average size of 50 nm (Fig. 1b). The corresponding X-ray diffraction (XRD) pattern (presented in Fig. 1c) suggests a face-centered cubic (fcc) structure (JCPDS No. 04–0783) of metallic Ag. After sulfidation, an octopod-like core@shell structure with a bulge sitting at each corner of the nanocube was formed (Fig. 1d,e). The diameter of the bulges is about 35 nm (the insert in Fig. 1e). From the transmission electron microscopy (TEM) image, distinct interfaces and different contrasts are found in the core@shell structures, suggesting that the structures contain multiple components. The XRD pattern of the core@shell structures (presented in Fig. 1c) reveals a minority of monoclinic Ag_2_S (JCPDS No. 14–0072) in addition to the majority of fcc-structured metallic Ag. In the high-resolution (HR) TEM image of a bulge (Fig. 1f), the lattice spacing of 0.24 nm are coincident with (121) plane of monoclinic Ag_2_S. Thus the lower contrast area in Fig. 1e (the bulges) is supposed to come from Ag_2_S, as the density of Ag_2_S (7.2 g·cm^−3^) is smaller than that of Ag (10.505 g·cm^−3^). Energy dispersive X-ray spectroscopy (EDS) analysis reveals that S element is mainly located at the corner positions, while Ag element is concentrated at central part of the core@shell structures (Fig. 1g and h). The results indicate that the octopod-like structure is an Ag@Ag_2_S core@shell structure with Ag_2_S bulges at the eight corners. As can be seen on the upper insert in Fig. 1e, the maximum shell thickness, which is located at the corners of the structure, is about 15 nm. The shell thickness gradually decreases towards the central part of the structure. The shell thickness at the central flat surface is about 5.5 nm (the lower insert in Fig. 1e). TEM images of a series of Ag@Ag_2_S core@shell structures have been presented in Fig. S1. The thickness at the central flat surface varies from a minimum value below 1 nm to a maximum value of 8 nm with the average thickness estimated to be 5 nm. The maximum shell thickness located at the corners of the structure varies from 13.1 nm to 18.2 nm, with the average value of 15.2 nm. The diameter of the Ag_2_S bulge varies from 30.7 nm to 38.5 nm, with the average value of 34.4 nm. Therefore, a desired core@shell structure with gradually varying shell thickness was successfully synthesized.

**Figure 2:**
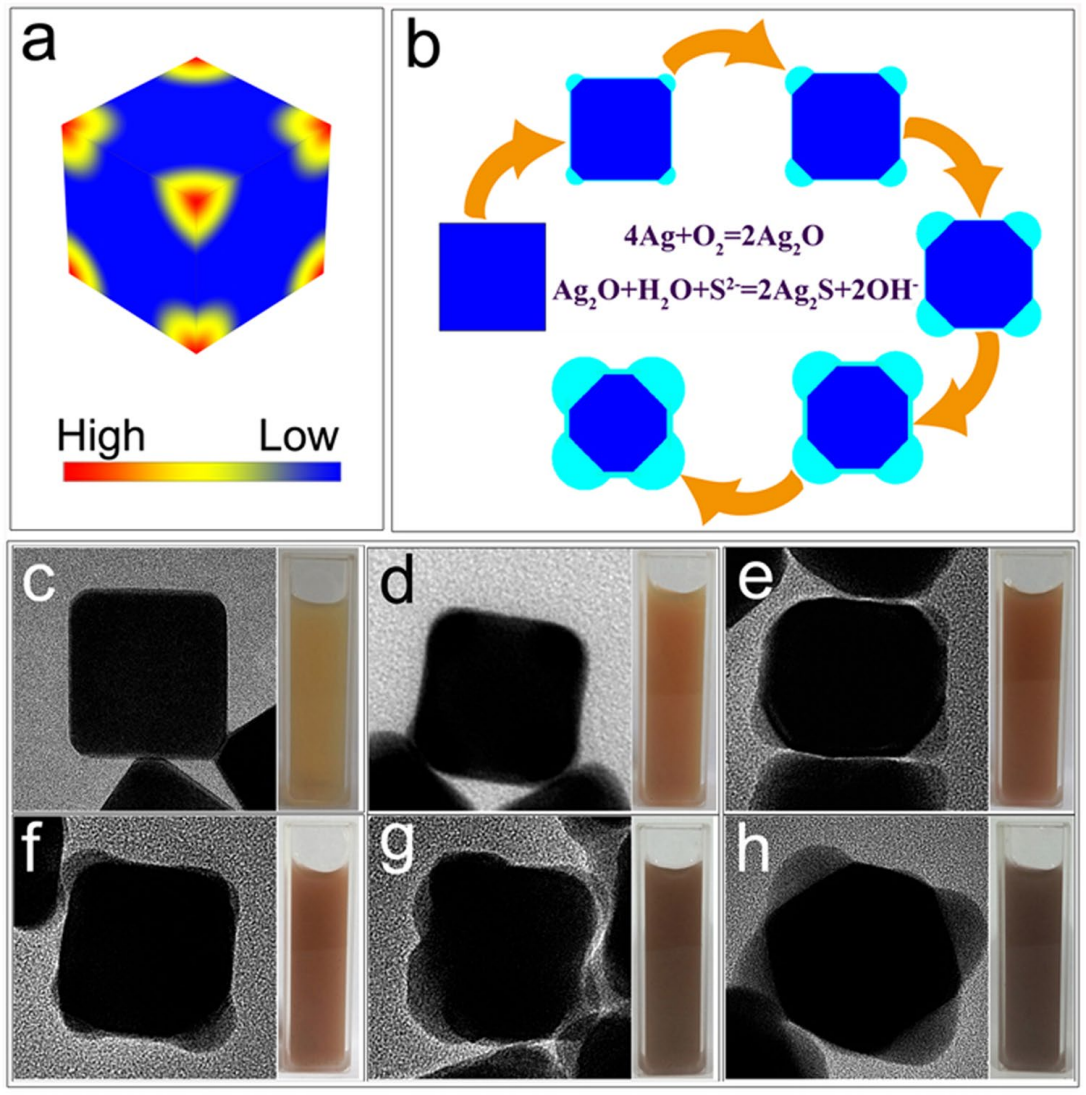
(**a**) Schematic illustrations of highly reactive areas for an Ag nanocube, (**b**) Schematic illustration of the evolution from an Ag nanocube to an Ag@Ag_2_S core@shell structure, and (**c**–**h**) The TEM images and colour evolution of the Ag@Ag_2_S core@shell structures at different reaction stages.

The cubic shape of the Ag templates plays the most important role in forming a core@shell structure with gradually varying shell thickness. The spatial difference in reactivity is the key reason for the gradually varying shell thickness. As it has been pointed out in the literatures^29,30^, the chemical reactivity is much higher at the sharp corners of the Ag nanocubes because of the high surface free energy at these areas. Therefore, the speed of oxidative etching (Ag^0^ → Ag^+^) is also higher at the sharp corners than the rest parts of the Ag nanocubes. Figure 2a is a schematic illustration showing the highly reactive areas of an Ag nanocube. The schematic illustration of the evolution from an Ag nanocube to an Ag@Ag_2_S core@shell structure is presented as Fig. 2b. At the right beginning of the process, Ag^0^ at the sharp corners of the nanocube is oxidized to Ag_2_O by the dissolved oxygen in the solution. The resulting Ag_2_O then reacts with S^2−^ (from the hydrolysis of TAA) to yield Ag_2_S. Given that the solubility product of Ag_2_S is extremely low (6.3 × 10^−50^ at 298 K), the amount of Ag^+^ diffusing into the solution is negligible. Sulfidation of the Ag nanocube starts from the sharp corners and spreads towards the central surface of the nanocube. It is worth to note that the reaction rate is higher at the corners than the central surface. It is also important to note that the molar volume of the resulting Ag_2_S region (33.9 cm^3^·mol^−1^) is much larger than that of the original Ag region (10.3 cm^3^·mol^−1^). These two reasons lead to the formation of Ag_2_S bulges bulging at the corners of the Ag nanocube. In contrast, the reaction rate at the central areas of the Ag nanocube is much lower, thus only a thin shell of Ag_2_S has also been formed at these areas. The idea can be verified by the experimental data. At the central area, the EDS analysis (Fig. 1e) shows a minor level of sulphur content which is lower than that at the corners. On the other side, silver content at the central area is higher than that at the corners. The results indicate that the Ag core is surrounded by an Ag_2_S shell with gradually varying thickness. The TEM images (Fig. 2c–h) of the Ag@Ag_2_S core@shell structures taken at different evolution stages agree well with the schematic illustration (Fig. 2b). The colour evolution of the suspensions, which indicates significant changes in the optical absorption characteristics of the resulting Ag@Ag_2_S core@shell structures, is also presented in Fig. 2. On the other hand, Ag@Ag_2_S nanocubes can also be synthesized by controlling the sulfidation degree of the Ag nanocube. At a lower degree of sulfidation, the difference in volume changes between the corners and the central flat surfaces is not significant, resulting in nearly identical shell thickness all over the Ag nanocube. At this stage, the product is Ag@Ag_2_S nanocube as previously reported in the literatures^15,25^.

### Broadband absorption of the octopod-like Ag@Ag_2_S core@shell structures

**Figure 3:**
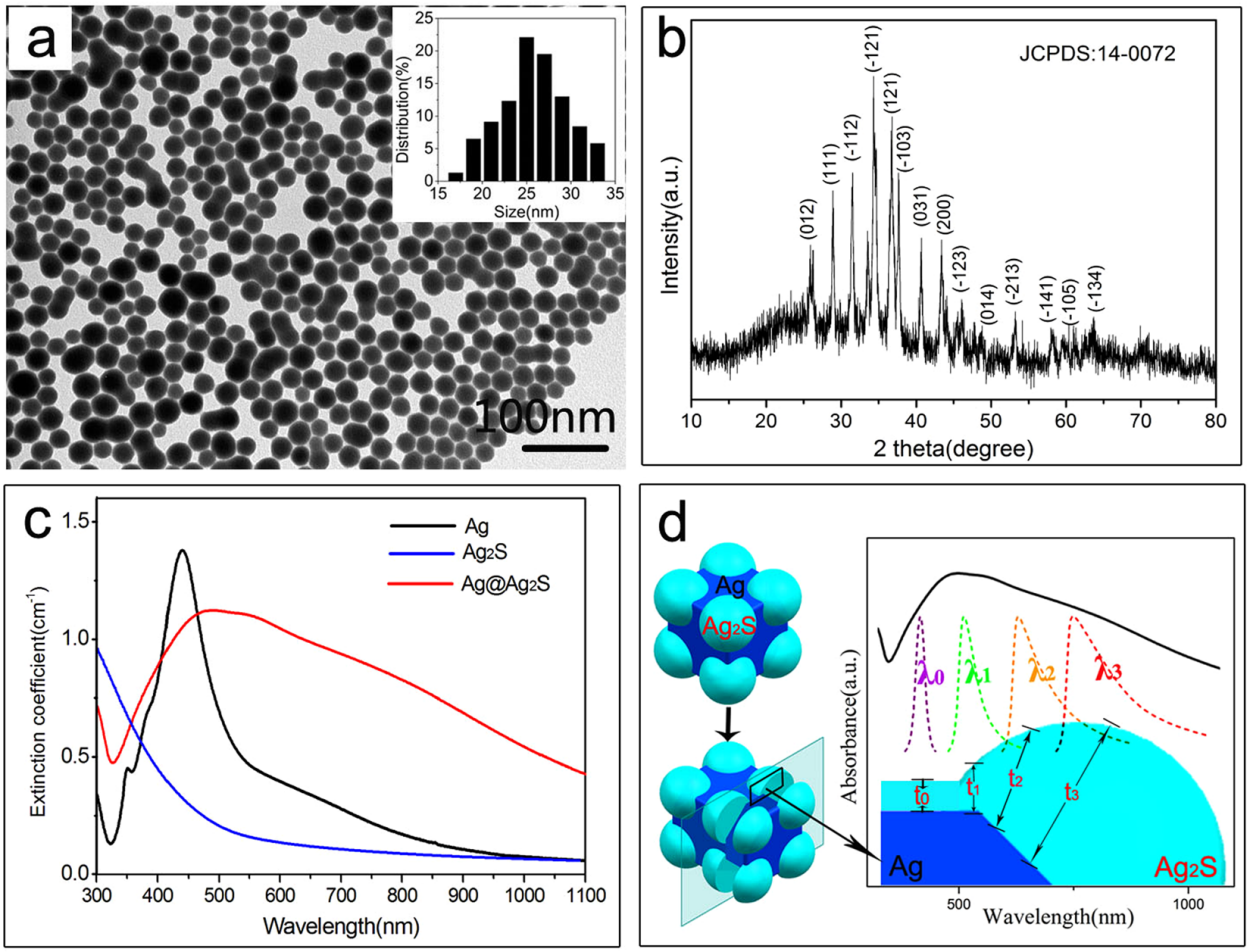
(**a**,**b**) The TEM image and XRD pattern of the Ag_2_S nanospheres, (**c**) The optical absorption spectra of the Ag@Ag_2_S core@shell structures, Ag nanocubes and Ag_2_S nanospheres. The spectra were recorded with 0.1 mg·mL^−1^ aqueous suspensions. (**d**) The model used to represent the Ag@Ag_2_S core@shell structures and schematic illustration of broadband absorption spectrum caused by varying shell thickness.

It is well known that the photothermal conversion property of materials is closely related to their optical absorption characteristics. To compare the optical absorption properties of the resulting core@shell structures to that of the corresponding shell materials (Ag_2_S), well dispersed Ag_2_S nanoparticles with average size of 25 nm have also been synthesized (Fig. 3a). The corresponding XRD pattern (Fig. 3b) reveals a monoclinic Ag_2_S phase (JCPDS No. 14–0072) which is identical to that of the shell materials. Figure 3c is the UV-Vis-NIR spectra recorded in transmission mode. To further evaluate the scattering effect, hemispherical transmittance was measured on a spectrophotometer equipped with an integration sphere^31^. The comparison of the hemispherical transmittance with the normal transmittance is presented as Fig. S1. The results show little difference between the hemispherical transmittance and normal transmittance of the suspension. By assuming that the difference between hemispherical transmittance and normal transmittance can represent the degree of scattering, we come to the conclusion that scattering is not significant. Therefore, the UV-Vis-NIR spectra can describe the absorption characteristics of the samples. As can be seen in Fig. 3c, the SPR absorption of the Ag nanocubes is centered at 450 nm. The extinction drops rapidly at wavelengths over 500 nm and becomes insignificant in the near infrared region. After sulfidation, the resulting octopod-like Ag@Ag_2_S core@shell structures exhibit a broadband absorption from 400 nm to 1100 nm. The extinction of the Ag_2_S nanoparticles is strong in the ultraviolet region but becomes negligible at wavelengths over 500 nm, which is in consistent with the reported data^32^. The results indicate that the octopod-like Ag@Ag_2_S core@shell structures show a novel optical property (broadband absorption), which neither the corresponding core material (Ag) nor shell material (Ag_2_S) possesses. This novel property is due to two main reasons. On the one hand, it has been reported that the plasmon wavelengths of metal nanostructures are sensitive to the dielectric properties of the surrounding medium^33,34^. The red-shift of the absorption spectra is attributed to the formation of Ag_2_S dielectric layer: the high refractive index (1.9–2.5) and relative dielectric constant (ε_r_ = 6) of Ag_2_S^24,29,30,35^. On the other hand, the broadband absorption of the octopod-like Ag@Ag_2_S core@shell structures is attributed to the gradually varying Ag_2_S shell thickness. As mentioned previously, the absorption wavelength is in direct relation to the shell thickness^22^. As illustrated in Fig. 3d, the shell thickness varies at different positions of the Ag@Ag_2_S core@shell structures. Based on the previous analysis, a specific shell thickness will lead to a specific absorption wavelength. For example, three different shell thicknesses, t_1_, t_2_ and t_3_, result in three different absorption wavelengths, λ_1_, λ_2_ and λ_3_, respectively. The absorption wavelength λ_0_ corresponds to the thickness at central flat surfaces of the octopod.

To better illustrate the difference in the absorption characteristics at different positions of the octopod, Finite-Difference Time-Domain (FDTD) simulation was conducted. The FDTD simulation was conducted based on a typical hybrid nanoparticle presented as the insert in Fig. 1e. Fig. S3a,b show the 2D electromagnetic (EM) spatial distribution for the Ag@Ag_2_S octopod with incidence (λ = 808 nm) perpendicular to the YOX plane (a) and the ZOX plane (b). The results show clearly that the EM response at the bulges of the octopod is much stronger than that at the central flat surfaces. Considering the random orientation of different Ag@Ag_2_S octopods, we further conducted the FDTD simulation at different typical incidence angles. The incidence wavelength varies from 600 nm to 1000 nm. The results are presented in Fig. S3c. The calculated absorb cross section shifts significantly as the incidence angle changes. Given that the absorption spectrum of the sample is the integrated intensity at different incidence angles, it can explain why a broad absorption peak is presented. Therefore, the octopod-like Ag@Ag_2_S core@shell structures having gradually varying shell thickness will certainly exhibit integrated absorption characteristics, just like the mixture of core@shell structures with different shell thicknesses. Owing to their broadband absorption property, the as-synthesized Ag@Ag_2_S core@shell structures may show significant PTC effects under the irradiation of lasers at different wavelengths. It is also worth to note that Ag_2_S has been found to exhibit efficient infrared emitting characteristics^36,37^ which enable the potential of our products in thermal sensing applications.

### Enhanced PTC properties of the octopod-like Ag@Ag_2_S core@shell structures

**Figure 4:**
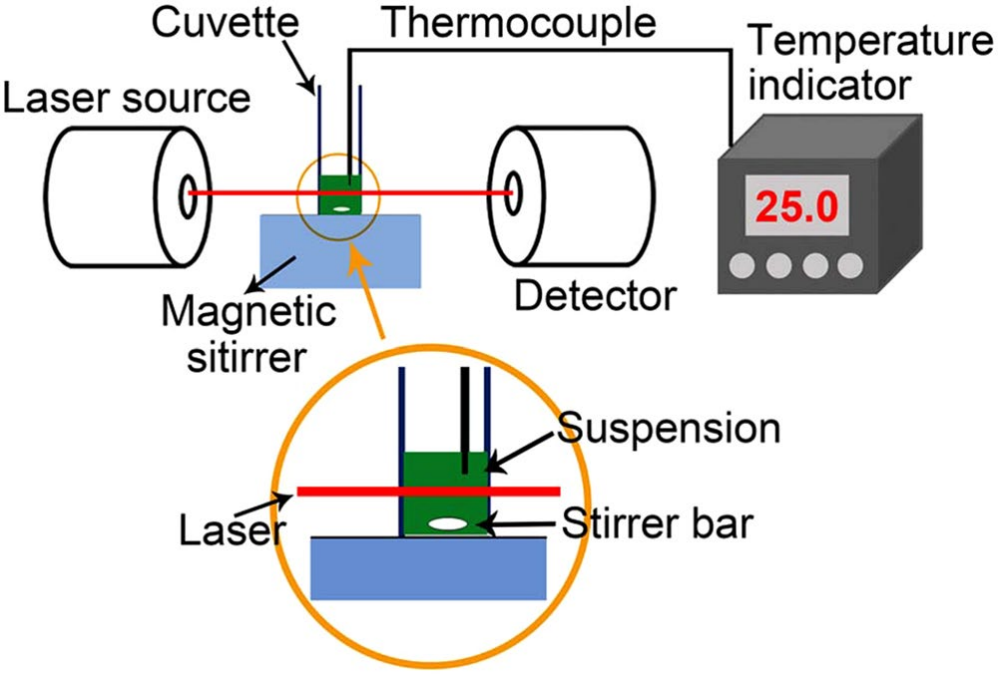
Schematic of the evaluation system for photothermal conversion properties.

**Figure 5:**
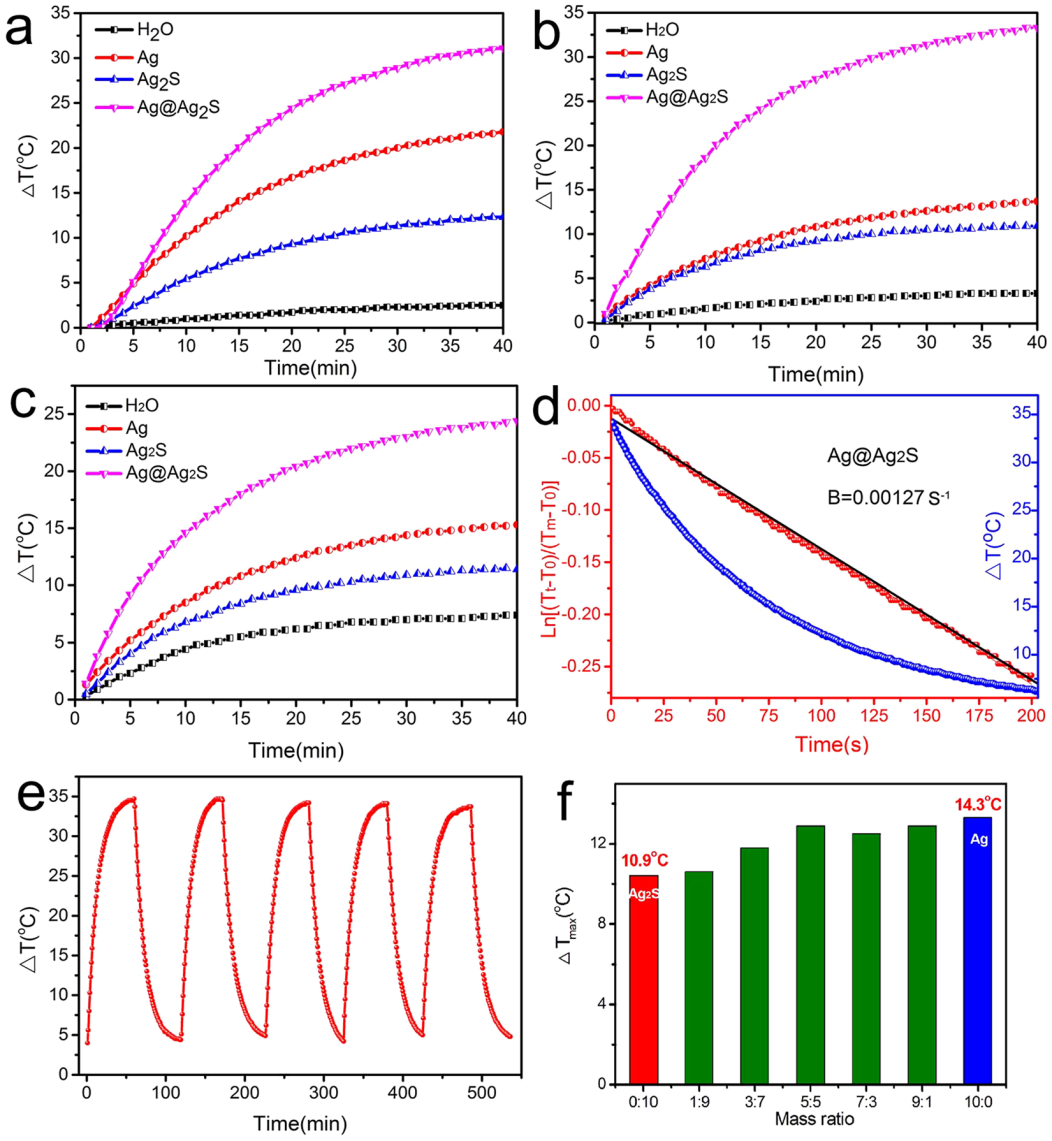
The temperature rises of the aqueous suspensions containing Ag@Ag_2_S core@shell structures, Ag nanocubes and Ag_2_S nanospheres under the irradiation of (**a**) 635 nm, (**b**) 808 nm and (**c**) 1064 nm lasers, (**d**) Plot of the natural logarithm of [(T_t_ − T_0_)/(T_m_ − T_0_)] as a function of time obtained from cooling stages after the laser was turned off, (**e**) The temperature rise of the aqueous suspensions containing Ag@Ag_2_S core@shell structures under the irradiation of an 808 nm laser over five laser-on/off cycles, and (**f**) The maximum temperature rises in the aqueous suspension containing Ag nanocubes and Ag_2_S nanospheres with different mass ratios under the irradiation of an 808 nm laser. The concentrations of the aqueous suspensions were all 0.1 mg·mL^−1^.

The PTC properties of the octopod-like Ag@Ag_2_S core@shell structures were tested on a lab-made evaluation system with water as reference. The PTC properties of the Ag nanocubes and the Ag_2_S nanoparticles were also tested for comparison. For a brief description, the samples were dispersed into deionized water to make aqueous suspensions. The suspension was then exposed to the irradiation of lasers. Three lasers with wavelengths of 635, 808, and 1064 nm were applied in the testing. The schematic diagram is presented as Fig. 4. For a typical test procedure, 1 mL suspension is loaded in a quartz cuvette and exposed to the illumination of the lasers. The power of the lasers is set to be 420 mW in order to get reasonable temperature rise in the suspension. After the laser is turned on, the temperature in the suspension will increase as a result of the PTC effect and finally became stable at a maximum temperature at which the heat generation from the PTC effect equals to the heat dissipation to the environment. Theoretically, the heat dissipation at the stable stage is proportional to the maximum temperature rise (difference between the maximum temperature and the ambient temperature). Therefore, the maximum temperature rise can be used to represent the PTC property of the sample. The profiles of temperature rise are presented in Fig. 5. In general, the temperature rise of the aqueous suspension containing Ag@Ag_2_S core@shell structures (0.1 mg·mL^−1^) is significantly higher than that of the aqueous suspensions containing the Ag nanocubes (0.1 mg·mL^−1^) and Ag_2_S nanoparticles (0.1 mg·mL^−1^). As a reference, water shows the lowest temperature rise. Under the irradiation of a 635 nm laser, the aqueous suspension containing Ag@Ag_2_S core@shell structures shows a maximum temperature rise of 32.9 °C. The corresponding temperature rises for the suspensions containing Ag nanocubes and Ag_2_S nanoparticles are 23.2 and 13.2 °C, respectively (Fig. 5a). The temperature rise for water is 3.3 °C. For the 808 nm laser, the maximum temperature rises for 1 mL aqueous suspensions containing Ag@Ag_2_S core@shell structure , Ag nanocubes, and Ag_2_S nanoparticles are 34.7, 14.3, and 10.9 °C, respectively. As a reference, the maximum temperature rise of 1 mL water is 3.3 °C (Fig. 5b). For the 1064 nm laser, the corresponding temperature rises for Ag@Ag_2_S core@shell structures, Ag nanocubes, Ag_2_S nanoparticles, and water are 24.4, 15.7, 11.5, and 7.4 °C, respectively (Fig. 5c). According to the previously reported method^38^, the PTC efficiency of the Ag@Ag_2_S core@shell structures (0.1 mg·mL^−1^ aqueous suspension) for 808 nm laser can be calculated to be 64.7% based on the data collected in the cooling stage (Fig. 5d). Likewise, the PTC efficiencies for 635 nm and 1064 nm lasers are determined to be 63.7% and 79.3%. It has been previously reported that the photothermal conversion efficiency of carbon nanotubes do not show significant change as the incident wavelength varies^39^. The wavelength-independency of photothermal conversion efficiency is an interesting phenomenon. It should be clarified that the PTC efficiency in the current work is defined by comparing the converted heat to the absorbed light and would not show significant dependence on wavelength. The gross efficiency, which is defined by comparing the converted heat to the incident light, is wavelength dependent because the absorption efficiency (defined as the fraction of the absorbed light to the incident light) is wavelength dependent. It should also be pointed out that the obtained efficiency can be substantially impacted by experimental conditions and determination methods as well. Therefore, there is evident discrepancy in the efficiencies of PTC materials. Despite the discrepancy, the efficiency of the Ag@Ag_2_S core@shell structures is comparable to the typical data reported previously. The typical data of efficiency are summarized in Table 1 together with the extinction and absorption cross section data. Based on these results, one can draw the conclusion that the as-synthesized Ag@Ag_2_S core@shell structures possess enhanced PTC property in a wide range of spectrum. Such property is attributed to the broadband absorption characteristics of the Ag@Ag_2_S core@shell structures and enables the products to become promising candidates as multi-functional PTC materials.

**Table 1:** The typical data concerning efficiency and optical absorption in the current work and the literature.

Materials	Efficiency (%)	Wavelength of laser (nm)	Extinction (cm^−1^)	Absorption cross section(m^2^)	Ref
Ag@Ag_2_S octopod	63.7 64.7 79.3	635 808 1064	1.01 0.82 0.46	1.2 × 10^−13^ 1.4 × 10^−15^ 8.0 × 10^−16^	this work
Au nanopolyhedrons	18	809	0.06	—	^20^
Au nanorods	62	815	—	0.3 × 10^−14^	^49^
MoS_2_ nanoflakes	27	808	0.88	—	^5^
Au-Ag_2_S hybrid	64	809	1.7	—	^20^
Au-ZnS hybrid	86	809	1.7	—	^20^
Au-Cu_7_S_4_ hybrid	63	980	—	—	^38^
Au-SiO_2_ hybrid	35	815	—	1.3 × 10^−14^	^49^
Au-Au_2_S hybrid	60	815	—	0.1 × 10^-14^	^49^
Au-Cu_2-x_Se hybrid	33	808	1.02	—	^50^
multi-walled carbon nanotubes	53	808	7.8	—	^39^

The Ag@Ag_2_S core@shell structures are also photothermal stable. As illustrated in Fig. 5e, the maximum temperature rise of the suspension containing the Ag@Ag_2_S core@shell structures shows little change after 5 cycles of photothermal heating and cooling within a time span of 500 min. The stability is attributed to the *in-situ* sulfidation strategy which results in clean and robust interface between the core and the shell of the Ag@Ag_2_S core@shell structure. To demonstrate that the enhanced PTC property cannot be achieved by simply mixing Ag nanocubes and Ag_2_S nanoparticles, maximum temperature rises for the aqueous suspensions containing both species at different ratios (1:9 to 9:1) were measured under the irradiation of an 808 nm laser (420 mW). The total concentrations of these two-component suspensions were all 0.1 mg·mL^−1^. For all ratios that involved, the maximum temperature rises vary between 10.6 to 14.3 °C (Fig. 5f), which are close to that of the suspensions containing Ag nanocubes and Ag_2_S nanoparticles, but significantly lower than that of the suspension containing Ag@Ag_2_S core@shell structures. Therefore, the enhanced PTC property can be better attributed to the novel structure in addition to the chemical composition. For nanostructures containing metallic component, localized surface plasmon resonance is the most frequently referred mechanism to explain the enhancement in photon absorption^20,40,41^. For the photothermal conversion mechanism of semiconductors^41–44^, it is proposed that heat is generated during the non-radiative de-excitation process due to phonon-assisted electronic decay and/or relaxation of free-carrier surface currents^41,44–46^. For hybrid nanostructures containing semiconductors, it is also pointed out that the presence of heterointerfaces usually induces the quenching of radiative de-excitation process and therefore enhances the non-radiative de-excitation process and delivers more heat^45^. On the other hand, coating metallic nanocrystals (like Ag nanocubes in the current work) with semiconductor materials (like Ag_2_S in the current work) can also enhance the photothermal conversion effect because of the additional light absorption channel^20^.

**Figure 6:**
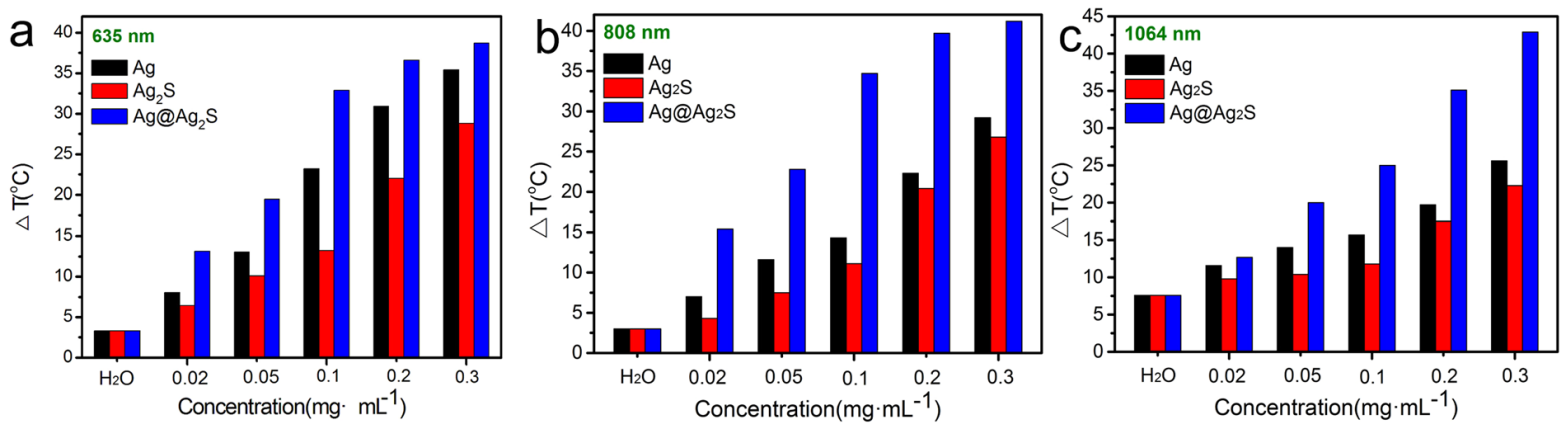
The influence of concentration on the maximum temperature rises in suspensions under the irradiation of lasers, (**a**) 635 nm, (**b**) 808 nm, (**c**) 1064 nm.

The maximum temperature rises for all suspensions can be further increased by increasing the concentrations. As presented in Fig. 6, higher maximum temperature rises correspond to higher concentrations of the suspension for identical power and wavelength of laser. At all concentrations, the suspensions containing the Ag@Ag_2_S core@shell structures show the highest temperature rise. Such properties enable the achievement of different temperature levels by adjusting the concentration of the suspension, and therefore can fulfil the requirement of different applications. In addition, the maximum temperature rises can also be adjusted by changing the laser power. As illustrated in Fig. S4, the maximum temperature rise of the suspension is linearly proportional to the laser power within the range in the current work.

## Discussion

We demonstrated the synthesis of octopod-like Ag@Ag_2_S core@shell structures with gradually varying thickness via an *in-situ* sulfidation method with Ag nanocubes as sacrificial templates. The as-synthesized core@shell structures exhibit broadband absorption characteristics and enhanced PTC properties in a wide range of spectrum. Taking the above example, we demonstrated a versatile strategy to design and synthesize nanostructures as multi-functional PTC materials. Such nanostructures have potential applications in multiple fields including photothermal therapy, solar energy collection and conversion, absorbing and stealth materials, and so on. The strategy can be applied to prepare different nanostructures with metallic cores other than the Ag@Ag_2_S core@shell structures in the current work, for the reason that it is based on well-defined physical mechanism. As previously mentioned, SPR absorption wavelengths of metallic surfaces can be significantly influenced by the species of the surrounding medium as well as the thickness of the medium. Based on the evidences presented in the literature, different thicknesses of the medium lead to different SPR wavelengths of the metallic cores. Therefore, nanostructures with gradually varying shell thickness will give integrated SPR wavelengths and thus exhibit broadband absorption characteristics. It is for sure that the thickness should vary in a reasonable range (within nanometer scale, for example). An ever increasing shell thickness may reach a turning point after which the increase in thickness will cease to be relevant and even lead to reverse effect.

We also demonstrated a strategy to synthesize heteromorphous core@shell structures by using sacrificial templates having spatially different reactivity. Sacrificial template methods are commonly used to synthesize nanostructures having similar shape and size as the templates. In the conventional applications of the sacrificial template methods, the templates usually have homogenous reactivity all over the surface. Interface chemical reactions may proceed at the same rate in all directions towards the cores. By controlling the reaction degree, one can synthesize core@shell structures with homogenous shell thickness or finally new species of nanospheres. In order to synthesize heteromorphous core@shell structures, however, templates having spatially different reactivity are essential. The Ag nanocube in the current work is just an example. One can choose the appropriate templates according to the desired shape, size, and species of the final products. It should be noted that the reaction medium is also important as it will influence the reaction rate significantly. Sulfidation of Ag nanocube in ethanol may result in homogenous thin shell of Ag_2_S, for the reason that the reaction rate in ethanol is quite low. On the contrary, too high reaction rate will lead to complete transformation from core materials to shell materials in very short time.

## Methods

### Materials and instrumentations

All chemicals were analytical reagents and used without further purification. The morphology and geometric structure of the as-prepared products were studied on a JEM-2000EX transmission electron microscope (TEM) and a field-emission scanning electron microscope (FESEM-6700). The phase composition of the as-prepared products was determined by X-ray diffraction (XRD) on a Rigaku D/MAX-2500/PC diffractometer. An Energy dispersive X-ray spectroscopy (EDS) analyser attached to a FEI Magellan 400 SEM was used to analyse the compounds and elemental distribution in the Ag@Ag2S core@shell structures. The optical absorption properties were investigated on a UV-vis-NIR spectrometer (Varian Cary 500).

### Synthesis of the Ag nanocubes

In a typical process, ethylene glycol (10 mL, EG) was added into a 50 mL round bottom flask and preheated in oil bath under magnetic stirring at 150 °C for 1 h. And then, polyvinyl pyrrolidone (PVP) solution in EG (2.5 mL, 20 mg·mL^−1^) was added into the heated EG solution. After 10 min, NaSH solution in EG (0.12 mL, 3 mM) was quickly added into the solution, followed by the addition of HCl solution in EG (1 mL, 3 mM) and CF_3_COOAg solution in EG (0.8 mL, 282 mM). The reaction was allowed to proceed for 1 h to obtain the Ag nanocubes, and then quenched by placing the flask in an ice-water bath with magnetic stirring for at least 10 min. The reaction solution was washed with acetone and deionized water to remove EG and excess PVP and re-dispersed into 40 mL deionized water to get Ag nanocubes aqueous suspension.

### Synthesis of the octopod-like Ag@Ag_2_S core@shell structures

In a typical process, 10 mL PVP aqueous solution (20 mg·mL^−1^) was added into 20 mL aforementioned Ag nanocubes aqueous suspension under magnetic stirring at room temperature. And then, thioacetamide (TAA) aqueous solution (10 mL, 0.2 mol·L^−1^) was added and stirred for 4 h. The reaction was quenched by centrifuging the solution at 10000 rpm for 10 min. The as-synthesized products were washed with deionized water and absolute ethanol successively and re-dispersed in deionized water for further characterization and measurement.

### Synthesis of the Ag_2_S nanoparticles

The Ag_2_S nanoparticles were prepared at ambient temperature. In the first step of a typical synthesis procedure, 42.5 mg PVP-K30 and 42.5 mg AgNO_3_ were dissolved in deionized water (50 mL) under magnetic stirring. After stirring for 30 min, TAA aqueous solution (0.0125 mmol) was added dropwise. The reaction was stopped after stirring for 30 min. The reaction solution was washed with deionized water and absolute ethanol for three times.

### Evaluation of the PTC properties

The PTC properties were studied on a lab-made evaluation system. Schematics of the evaluation system are presented as Fig. 4. The system was equipped with solid state lasers supplied by Aunion Tech. Co., Ltd., Shanghai, China. In a typical procedure, 1 mL suspension was loaded in a quartz cuvette with a chamber of 4 × 1 × 1 cm to be tested. A thermocouple with an accuracy of ±0.1 ^o^C was inserted into the suspension and connected to a computer. It should be noted that the thermocouple was keep away from the direct hitting of the laser beam. When the incident laser beam was allowed to hit the suspension, the temperature in the suspension increased gradually. After irradiating for a period of time, the laser beam was blocked and the temperature variation in the suspension during the whole process was detected and recorded for analysis.

The heating model is similar to the ones previously published^47,48^. The energy balance eq. 1 was listed as followed. It can be determined by the rate of heat generation (${Q}_{IN}$) and the heat dissipation to the environment (${Q}_{OUT}$).

1 $${m}_{w}{c}_{w}\frac{dT}{dt}={Q}_{IN}-{Q}_{OUT}\,$$

where ${m}_{w}$ and ${c}_{w}$ are the mass and specific heat capacity of water, respectively. *T* is the temperature and *t* is time. It should be noted that, in our system, the mass and specific heat capacity of the nanoparticles are negligible because they are much smaller than that of water.

The heat generation *Q*_*IN*_ is showed as eq. 2,

2 $$\,{Q}_{IN}=({P}_{IN}-{P}_{TRA})\eta $$

where $\eta$ is the photothermal conversion efficiency of the suspension. *P*_*IN*_ is the power of the incident laser (420 mW). *P*_*TRA*_ is transmission power of the laser, which is detected on spot by an Optical Power and Energy Meter (THORLABS, PM100D).

The heat dissipated to the environment is showed as eq. 3.

3 $${Q}_{OUT}=hS({T}_{t}-{T}_{0})$$

where *h* is a constant independent to $T$, *S* is the surface area of the cross-sectional area perpendicular to conduction. *T*_*T*_ is the temperature of the suspension at time $t$, *T*_*0*_ is the ambient temperature. By defining $\,{\rm{\Delta }}T={T}_{t}-{T}_{0}f$, eq. 1 can be expressed as

4 $$\frac{d\Delta T}{dt}=\frac{({P}_{IN}-{P}_{TRA})\eta }{{m}_{w}{c}_{w}}-\frac{hS({T}_{t}-{T}_{0})}{{m}_{w}{c}_{w}}$$

At the cooling stage after the laser is turned off,$\,{Q}_{IN}=0$, and eq. 4 can be written into

5 $$\frac{d\Delta T}{dt}=-B({T}_{t}-{T}_{0})$$

where we define $B=\frac{hS}{{m}_{w}{c}_{w}}$ as the rate constant associated with heat loss, which can be determined by measuring the decreasing temperature profile after laser is turned off (Fig. 5d). And the temperature trace is showed as eq. 6, where ${T}_{m}\,\,$is the maximum temperature.

6 $$\mathrm{ln}\,\frac{{T}_{t}-{T}_{0}}{{T}_{m}-{T}_{0}}=-Bt$$

At thermal equilibrium, where $\,{Q}_{IN}={Q}_{OUT}$, eq. 4 equals zero, and the photothermal conversion efficiency can be calculated from

7 $$\eta =\frac{B{m}_{w}{c}_{w}({T}_{m}-{T}_{0})}{{P}_{IN}-{P}_{TRA}}$$

### Data availability statement

Materials and data are available from the corresponding author at htzhu1970@163.com and dxwu100@163.com.

## Electronic supplementary material

Supplementary information
