# Supplementary material for: Broadband absorption and enhanced photothermal conversion property of octopod-like Ag@Ag2S core@shell structures with gradually varying shell thickness
Source: Sci Rep. 2017 Dec 19;7:17782. doi: 10.1038/s41598-017-18220-1 (PMC5736611; doi:10.1038/s41598-017-18220-1)
Supplement: Supplementary file 1 — Supplementary information [file 41598_2017_18220_MOESM1_ESM.pdf]

Supplementary information

Broadband absorption and enhanced photothermal conversion property of octopod-like Ag@Ag_2S core@shell structures having gradually varying shell thickness

Qian Jiang[1], Wenxia Zeng[1], Canying Zhang[1], Zhaoguo Meng[2], Jiawei Wu[3], Qunzhi Zhu[3], Daxiong Wu[1*] and Haitao Zhu[1*]

1) College of Materials Science and Engineering, Qingdao University of Science and Technology, Qingdao, Shandong, 266042, P. R. China.

2) College of Electromechanical Engineering, Qingdao University of Science and Technology, Qingdao, Shandong, 266042, P. R. China.

3) College of Energy and Machenical Engineering, Shanghai University of Electric Power, Shanghai, 200090, P. R. China.

* To whom correspondence should be addressed:

Professor Haitao Zhu

Tel: +86 532 84022676;

Fax: +86 532 84022814;

E-mail: htzhu1970@163.com

Dr. Daxiong Wu

E-mail: dxwu100@163.com

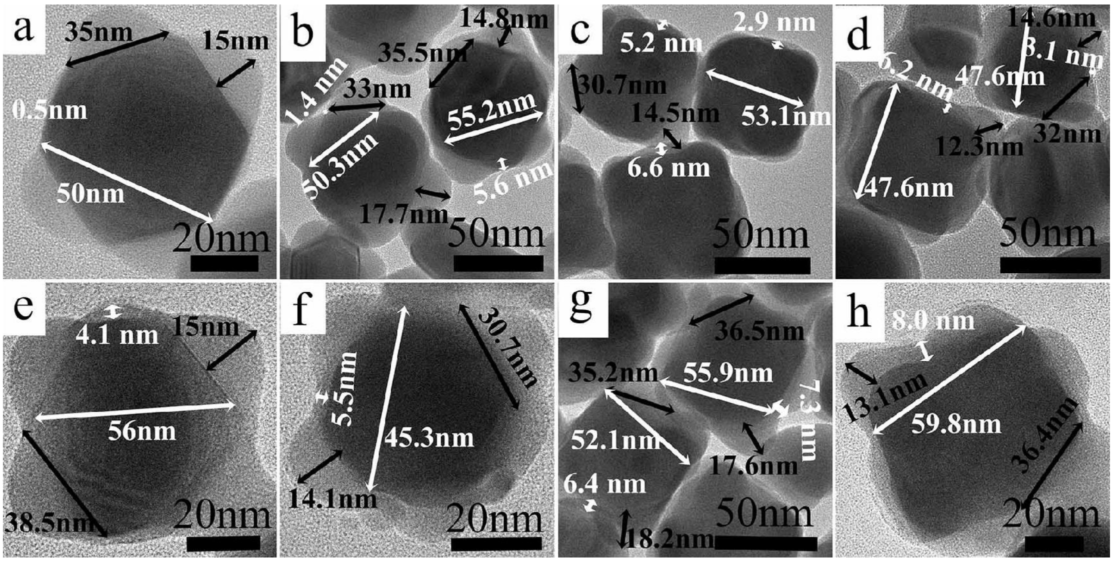

Figure S1. TEM images of the $Ag@Ag_2S$ core@shell structures.

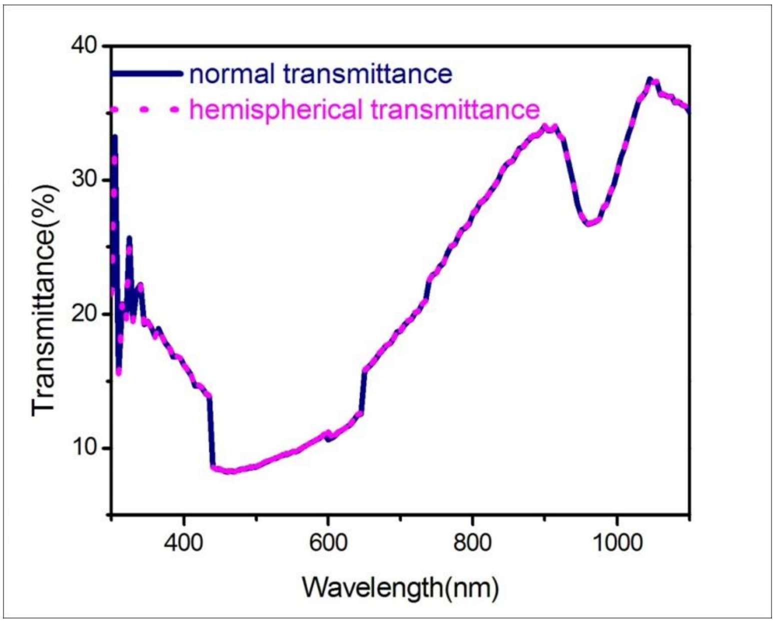

Figure S2. The hemispherical transmittance and normal transmittance of the suspension containing $Ag@Ag_2S$ core@shell structures (0.1 $mg \cdot mL^{-1}$).

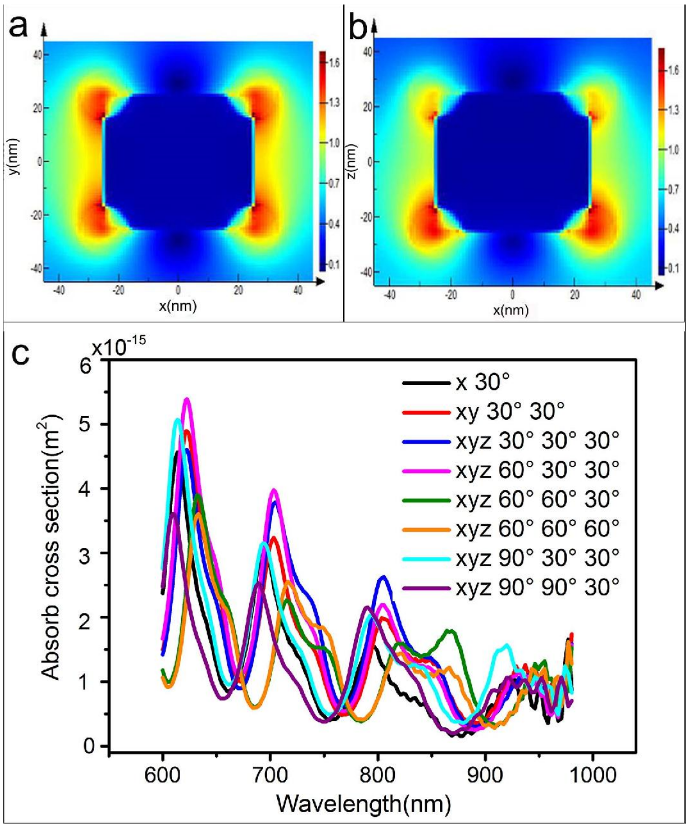

Figure S3. The FDTD simulated results of the $Ag@Ag_2S$ core@shell structure. (a-b) The 2D electromagnetic (EM) spatial distribution of the $Ag@Ag_2S$ core@shell structure. The Ag core in the core@shell structure is 50 nm in size. The Ag_2S shell thickness at the central flat surface is 5 nm and the maximum shell thickness at the corner is 15 nm. The diameter of the Ag_2S bulge is 35 nm. The parameters (n and k) for Ag come from the database of the simulation software. The parameters (n and k) for Ag_2S are adopted from reference S1. (c) The calculated absorb cross section at different incidence angles.

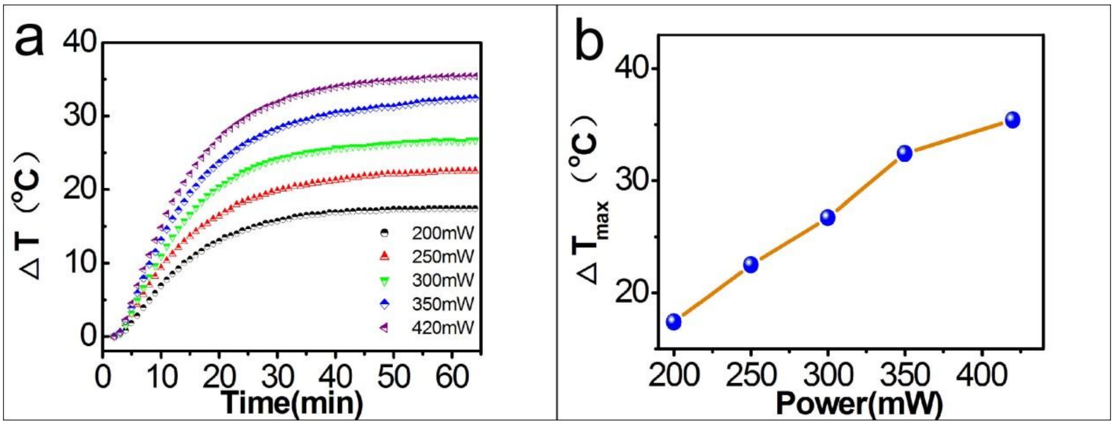

Figure S4. The temperature profiles (a) and the maximum temperature rise (b) of the suspension containing the Ag@Ag_2S core@shell structures (0.1 mg·mL^{-1}) under the irradiation of an 808 nm laser at different power.

References

S1. FI Ezema. et al. Growth and optical properties of Ag_2S thin films deposited by chemical bath deposition technique. *J. Chem. Technol. Metall.* **42**, 217-222 (2007).
